# Estimation of Complexity of Sampled Biomedical Continuous Time Signals Using Approximate Entropy

**DOI:** 10.3389/fphys.2018.00710

**Published:** 2018-06-11

**Authors:** Luca Mesin

**Affiliations:** Mathematical Biology and Physiology, Dipartimento di Elettronica e Telecomunicazioni, Politecnico di Torino, Turin, Italy

**Keywords:** complexity, approximate entropy, EEG, time series embedding, signal prediction

## Abstract

Non-linear analysis found many applications in biomedicine. Approximate entropy (ApEn) is a popular index of complexity often applied to biomedical data, as it provides quite stable indications when processing short and noisy epochs. However, ApEn strongly depends on parameters, which were chosen in the literature in wide ranges. This paper points out that ApEn depends on sampling rate of continuous time signals, embedding dimension, tolerance (under which a match is identified), epoch duration and low frequency trends. Moreover, contradicting results can be obtained changing parameters. This was found both in simulations and in experimental EEG. These limitations of ApEn suggest the introduction of an alternative index, here called modified ApEn, which is based on the following principles: oversampling is compensated, self-recurrences are ignored, a fixed percentage of recurrences is selected and low frequency trends are removed. The modified index allows to get more stable measurements of the complexity of the tested simulated data and EEG. The final conclusions are that, in order to get a reliable estimation of complexity using ApEn, parameters should be chosen within specific ranges, data must be sampled close to the Nyquist limit and low frequency trends should be removed. Following these indications, different studies could be more easily compared, interpreted and replicated. Moreover, the modified ApEn can be an interesting alternative, which extends the range of parameters for which stable indications can be achieved.

## Introduction

Some biomedical data have non-linear properties and complex behavior, e.g., heart rate variability (Lake and Moorman, 2011; Liu et al., 2011), electroencephalogram (EEG; Mesin and Costa, 2014; Li et al., 2016), electromyogram (EMG; Mesin et al., 2016), pupillogram (Mesin et al., 2013; Onorati et al., 2016), center of mass during quiet standing (Liang et al., 2017). The estimation of their level of regularity can be useful in many applications (Seely and Christou, 2000; Gao et al., 2015). Signal complexity is usually estimated assuming that the data were generated by a dynamical system in stationary conditions (i.e., ruled by constant deterministic laws) which can possibly develop irregular behavior due to its non-linearity (Kantz and Schreiber, 1997). However, biomedical data are often non-stationary. Thus, they are usually split into short time intervals in which the physiological system can be assumed in (quasi) stationary conditions. Using such short epochs, the estimation of data regularity is not easy, as the dynamics of the system cannot be explored completely. Moreover, biomedical data are also noisy. For these reasons, the approximate entropy (ApEn) was introduced (Pincus, 1991) as an index which could provide a rough, but quite stable indication of complexity even from short and noisy epochs.

Approximate entropy depends on parameters, which are usually chosen with large ranges in the literature. The main parameters to be selected are the threshold *r* under which a recurrence is found (also called filtering level, Pincus et al., 1991, or tolerance, Richman and Moorman, 2000), the length of the runs of data *m* (or embedding dimension) and the duration of the epoch *T*. A further important parameter when studying continuous signals is the sampling frequency *f*_s_: usually, poor care is given to it in the literature (it is only important that Nyquist limit is satisfied), but it has great effect on ApEn estimation, as discussed in the following. A few examples of results discussed in the literature on EEG and corresponding parameters are listed below.

• Lower complexity of scalp EEG were obtained in patients with schizophrenia than in controls, using *f*_s_ = 250, *r* equal to one fourth of the standard deviation of the signal (std), *m* = 10 and *T* = 20 s (Akar et al., 2016). Using different techniques, both increased and decreased complexity was documented on schizophrenic patients with respect to controls (Fernández et al., 2013; probably these contradicting results depend on the effect of factors like medication, symptomatology and age).
• ApEn of EEG was found to be lower in Alzheimer patients than in controls in the parietal region (Abásolo et al., 2005; data sampled at 256 Hz after limiting the bandwidth to 40 Hz, *r* = 0.2std, *m* = 1, *T* = 5 s).
• ApEn of EEG from adolescents with attention-deficit/hyperactivity disorder was significantly lower than in controls over the right frontal region during a cognitive task, but not at rest (Sohn et al., 2010; EEG sampled at 250 Hz after limiting the bandwidth to 50 Hz, *r* = 0.2std, *m* = 3, *T* = 20 s). ApEn also decreased in case of lower degree of cognitive activity (Natarajan et al., 2004; EEG sampled at 500 Hz after limiting the bandwidth to 50 Hz, *r* = 0.15 std, *m* = 2, *T* not specified, only the duration of the dataset, i.e., 20 min, was indicated).
• ApEn estimated from EEG showed variations among genders, suggesting differences in coding information (Jausovec and Jausovec, 2010; EEG sampled at 1 kHz after limiting the bandwidth to 70 Hz, but different low frequency rhythms were then studied, so that data could be sampled also at more than 100 times the bandwidth; *r* = 0.15 std, *m* = 2, *T* = 4 s).
• ApEn was found to decrease when estimated from EEG pre and post the application of stressors, i.e., Stroop test and sleep deprivation (Alonso et al., 2015; bandwidth limited to 35 Hz and signal re-sampled at 100 Hz, *m* = 3, *T* not indicated and *r* chosen in order to get the maximum value of ApEn, as suggested by Lu et al., 2008).
• ApEn allowed to identify six different stages of consciousness during sleep (Burioka et al., 2005; EEG sampled at 200 Hz after limiting the bandwidth to about 33 Hz, *r* = 0.2 std, *m* = 2, *T* = 10 s). An adaptive approach was applied in (Maen and Tadanori, 2013), where large ranges of parameters were explored and the discrimination of wakefulness states among drowsiness and fully awakening was optimal studying alpha waves of EEG sampled at 200 Hz (thus, sampling at about 16 times the bandwidth), with *r* equal to either 0.5 std or 0.95 std, *m* equal to either 8 or 9, *T* of either 0.25 or 0.5 s. On the other hand, ApEn of EEG recorded from subjects driving while sleep deprived showed no significant changes preceding driving errors (Papadelis et al., 2007; EEG sampled at 200 Hz, *T* = 1 s; *m* and *r* were not indicated for ApEn, but for cross-ApEn they were 1 and 0.2 std, respectively, so that these values were probably used also for estimating ApEn).
• ApEn of EEG was lower in patients in persistent vegetative state, than in case of minimally conscious state, than in controls (Wu et al., 2011; EEG sampled at 500 Hz after limiting the bandwidth to 100 Hz, *r* = 0.2 std, *m* = 2, *T* = 8 s).
• Monotonic variation of ApEn of EEG was found in relation with anesthetic induction (Koskinen et al., 2006; EEG sampled at 200 Hz after limiting the bandwidth to 40 Hz, *r* chosen in the range 0.05–0.5 std or fixed, *m* = 2, *T* chosen in the range 5–15 s). On the other hand, ApEn did not correlate with the depth of anesthesia (Jordan et al., 2006; EEG sampled at 1 kHz, duration of epochs 10 s, parameters for ApEn estimation not indicated). The ApEn of EEG during various depths of sedation showed opposite behaviors in (Ferenets et al., 2006), when different filters or parameters were tested (EEG sampled at 400 Hz after limiting the bandwidth to 19, 37, or 47 Hz, *r* chosen as either 0.1 or 0.2 std, *m* = 2,…,6, *T* chosen in the range 5–60 s): specifically, the value of ApEn was usually decreasing with the depth of sedation, but it was reversed, especially in the region of lighter sedation, if high frequencies were cut off and the delta frequency band was fully incorporated; moreover, in the case of short epochs, higher ApEn was found during deeper sedation if the embedding dimension was high.
• ApEn allowed to localize epileptic foci from EEG sampled at 200 Hz, using *r* = 0.5 std, *m* = 2, *T* = 10 s (Zhen et al., 2013). Using intracranial EEG at the focal locations, during seizures ApEn was found to increase (Abásolo et al., 2007; sampling rate 256 Hz after limiting the bandwidth to 40 Hz, *r* = 0.25std, *m* = 1, *T* = 10 s), but also to drop (Srinivasan et al., 2007; sampling rate 173.61 Hz after limiting the bandwidth to 40 Hz, *r* chosen in the range 0.1–0.9 std, *m* chosen in the range 1–3, *T* in the range 1–11.8 s).
• ApEn allowed to predict the onset of focal epileptic seizures with a mean prediction time of about 25 min processing EEG using *r* = 0.2 std, *m* = 2, *T* = 5 s (Zhen et al., 2014; sampling frequency not specified; the method was tested by the author with available data from both adults and children, without obtaining statistically different values of ApEn during pre-ictal and inter-ictal epochs).
• Good classification performances were obtained in a BCI application (Gupta et al., 2014) applying ApEn to EEG sampled at 128 Hz, with *m* selected in the range 1 to 3 and *r* in the range 0.1–0.6 std (*T* was not specified).

Even if considerable results were obtained in the literature on the application of ApEn on EEG, the large range of adopted parameters impedes the comparison of different works. Moreover, the author found some problems in replicating these successful results when using his own data. There is the suspect that (even when it is not clearly stated) parameters have been chosen adapting to the data in order to get specific outcomes, e.g., a good differentiation of different groups. However, this could lead to possible over-fitting of the available signals, which could reflect into problems in replicating the results using other data^1^. Moreover, by this method, discrimination is achieved, but there is no guarantee that the group with larger ApEn is also characterized by higher complexity. This could be source of possible contradicting results in the literature (as the above-mentioned references Abásolo et al., 2007; Srinivasan et al., 2007, obtaining an increase and a decrease of ApEn during epileptic seizures, respectively; moreover, Koskinen et al., 2006 supports that ApEn of EEG is related to the level of sedation, whereas Jordan et al., 2006 indicates that it is not). Furthermore, when investigating a new problem, it is not simple to predict the effect of changing a specific parameter (and the interaction with the other parameters), so that the optimal selection could be based on either a trial-and-error approach or an exhaustive search. However, by selecting the parameters after many tests, there is the risk that they are finally chosen to force the data to show what expected or that only positive outcomes (significant and in line with the literature) are shared with the scientific community. Thus, there is the need of defining some criteria for parameter selection in order to compare different studies, increase their repeatability and help to progress in the study of complexity by the use of ApEn. Consider also that the mathematical meaning of ApEn as an index of complexity could even fail when parameters are out of specific ranges. Furthermore, notice that, in the literature, the significance of the estimations of ApEn is usually not tested with surrogates (Schreiber and Schmitz, 1996; Mesin, 2017), so that it is not clear if the shown results reflect the assumed non-linearity of the data.

In this paper, an interpretation of ApEn is provided and the effect of changing parameters is explored. This investigation leads to the proposal of some rules to choose properly the parameters for the estimation of ApEn and to the introduction of a new index, which is a modification of ApEn.

## Materials and Methods

### Introduction

ApEn quantifies the complexity/unpredictability of fluctuations in a time series. Intuitively, the presence of repetitive patterns of fluctuation in a time series renders it more predictable (and hence less complex) than another in which such patterns are absent. Indeed, the future dynamics of the data could be forecasted based on that of previous patterns similar to the considered one (Farmer and Sidorowich, 1987).

ApEn expresses the logarithmic likelihood that the signal repeats itself within the tolerance of *r* both for *m* and for *m*+1 points. In this way, it approximates the estimation of entropy, which is the rate of information production (Eckmann and Ruelle, 1985). Its definition requires the steps detailed below, which can be interpreted within the framework of time series embedding and prediction (Kantz and Schreiber, 1997). Notice that the proposed interpretations hold for a sampled continuous time signal. Thus, discrete datasets (with heterogeneous sampling rate), which could be interpreted as Poincaré maps of continuous processes (Kantz and Schreiber, 1997), are not considered here. However, ApEn found many important biomedical applications to such data, like in the fields of neuronal spiking activity (Yang et al., 2002) and heart rate variability (Balasubramanian et al., 2017, even if some sensitivity on parameters was also found in Karmakar et al., 2017).

### Definition and Interpretation of ApEn

The scalar time series is embedded into a phase space of vectors (also called runs or templates) of delayed coordinates (or phases)

(1) $$X(i)=[x(i)\begin{array}{llll} & x(i-1) & \ldots & x(i-m+1)] \end{array}$$

where *x(i)* is the *i*th sample of the investigated time series x(⋅) and *m* is the embedding dimension (Kantz and Schreiber, 1997; Mesin, 2017). The correlation integral indicates the probability that the embedded vector *X(i)* is similar to other vectors within a tolerance *r*

(2) $$\begin{array}{cc} C_{i}^{m}(r)=\frac{N_{i}^{r}}{N-m+1}, & i=1,\;\ldots ,N-m+1 \end{array}$$

where *N* is the number of samples of the data and ${\text{N}}_{\text{i}}^{r}$ is the number of vectors with distance from *X(i)* smaller than *r*. As definition of distance, the *L*_∞_ norm was chosen (i.e., the maximum distance between pairs of elements of the two vectors). The vectors *X*(n), where *n* indicates the arbitrary sample running on time, describe a trajectory in the phase space. Thus, the definition of correlation integral $C_{i}^{m}(r)$ requires to count the number of recurrences $N_{i}^{r}$ of the trajectory to points close to *X(i)* and to divide by the number of possible pairs (thus, estimating the percentage of neighboring points of *X(i)* or the probability that the trajectory has recurrences close to it). The study of recurrences (also called neighboring points or matches) is very important in the non-linear analysis of time series and lead to the definition of many indexes of complexity and non-linearity (Webber and Zbilut, 1985; Kantz and Schreiber, 1997).

Finally, ApEn is defined based on the correlation integral, computed for two embedding dimensions

$$ApEn(m,r,N)=\phi^{m}(r)-\phi^{m+1}(r)$$

where

(3) $$\phi^{m}(r)=\frac{1}{N\text{​}-m+1}\sum_{i=1}^{N-m+1}\ln\;C_{i}^{m}(r)$$

The number of recurrences is higher in the lower dimension. Indeed, by increasing the dimension from *m* to *m*+1, one element is added to the vectors. This means that recurrences in dimension *m*+1 are also recurrences in dimension *m*, but it could happen that two vectors which are close in dimension *m* are not neighbors in dimension *m*+1, which means that the last added elements of the two vectors are more distant than the tolerance *r*. Thus, the reduction of the number of recurrences is related to the divergence of trajectories (that were close in dimension *m*, but not when adding an additional sample). Such a divergence is a marker of complexity and an indication of low predictability of the time series (measured also by other non-linear indexes, like Lyapunov exponent, Kantz, 1994, or determinism in recurrence quantification analysis, Webber and Zbilut, 1985). This observation justifies the fact that ApEn is larger when the probability that the trajectories diverge is greater: indeed, the logarithm in the definition of ϕ^m^(r) is monotonic, so that ApEn increases if the number of recurrences decreases when the embedding dimension is increased (from *m* to *m*+1).

### Expected Problems in Using ApEn

Notice that the study of the divergence of the trajectory in the phase space is feasible only if the embedding dimension is large enough to rule out the false near neighbors (Kantz and Schreiber, 1997). Indeed, when *m* is small, the trajectory may show intersections or recurrences due to its projection in a low dimensional space. The dynamics of the system around false neighbors (i.e., the possible divergence of trajectories passing through them) is obviously not related to the complexity of the system. Thus, the inclusion of false recurrences can bias the estimation of ApEn.

S. M. Pincus, the inventor of ApEn, suggested to use a small value of embedding dimension *m* (usually *m* = 2 or 3), an epoch duration of at least 10*^m^*–20*^m^* samples and a tolerance *r* equal to the 20% of the standard deviation of the signal (Pincus and Goldberger, 1994). However, as mentioned in the “Introduction”, wide ranges of parameter values were considered in applications. The following considerations suggest that parameters should be chosen carefully in order that the information extracted by ApEn is reliable and that possible problems (tested in the following) are avoided.

(1) ApEn is sensitive to the sampling frequency. As the delay between phases is fixed to be 1, over-sampling a signal corresponds to a linearization, which is expected to reduce ApEn. Indeed, it is simpler to predict the subsequent sample of a time series if data are over-sampled, as the new sample is close to the previous ones. Thus, the number of recurrences remains about constant in different embedding dimensions (the trajectory has not enough time to diverge) and the time series appears to be more predictable and hence less complex. Notice also that the maximum value of ApEn is ln(N - m) which is enlarging as the sampling frequency increases (as *N* becomes larger if the epoch duration is fixed); thus, the estimation of ApEn relative to its maximum possible value is further decreased by over-sampling the data.
  In the applicative papers quoted in the Introduction, a filter usually selected a specific bandwidth of EEG. On the other hand, if only an anti-aliasing filter is used, the over-sampled data include high frequency noise. In such a case, the divergence of a recurrent point is related to the noise, more than to the deterministic dynamics of the trajectory in the phase space. Thus, in such a condition, ApEn is expected to provide an estimation of the complexity of the noise, failing to decode the determinism of the system.
  To remove this problem, the data should be sampled close to the Nyquist limit or the delay between samples of embedded vectors should be larger than 1, equal to *f*_s_/*f*_N_, where *f*_N_ is a frequency close to the Nyquist limit (e.g., *f*_N_ could be chosen as three times the bandwidth of the time series).
(2) Fixing the tolerance *r*, the probability of finding recurrences decreases if the embedding dimension *m* is increased (notice that *m* was as large as 10 in some papers in the literature, even if, as mentioned above, it was suggested that samples in an epoch should be at least 10*^m^*–20*^m^* for a reliable estimation of ApEn, Pincus and Goldberger, 1994). Some considerations can be given to justify the relation between the embedding dimension and the number of samples needed to study reliably the time series. For simplicity, assume that the data is a set of independent, uniformly distributed samples, so that their range is $2\sqrt{3}\approx 3.5$ times the std. If *r* is 0.2 std (as suggested in Pincus and Goldberger, 1994), there are about 17.5 segments of length *r* with equal probability of finding recurrent points within them. If the time series is embedded in a space of dimension *m*, the number of boxes in which to find recurrences is about 17.5*^m^*. As each recurrent point should have many neighbors, e.g., *M* = 10 neighbors, to get reliable statistical conclusions (and hence a stable estimation of ApEn), the duration of the epoch (in terms of number of samples) needed to find sufficient recurrences to compute reasonably ApEn is *M*⋅17.5*^m^* (which becomes rapidly huge as the embedding dimension increases). The previous example should be considered as a worst case, as deterministic trajectories are usually attracted on a portion of the phase space with dimension that is less than *m* (Kantz and Schreiber, 1997). However, empirical trials indicate that, considering short epochs (with an order of 100–1000 samples) and *r* = 0.2 std, it is easy to get only few recurrences when *m* is about 3 or 4 (unless the data are quasi-periodic, linear, or over-sampled), in line with Pincus and Goldberger, 1994. In such a case, the measure of complexity or predictability (related to the percentage of neighbors that remain close to each other when extending the dimension) is not statistically feasible. When the number of recurrences is very low, there is even the possibility that, due to the different denominators in the definitions of ϕ^m^(r) and ϕ^m+1^(r), negative values of ApEn are obtained.
(3) The logarithm in the definition of entropy gives a larger weight to rare events (i.e., with a few recurrences), as they are more informative. Using short epochs, the estimation of ApEn could become quite unstable, as it depends on the behavior of rare events that are poorly represented.
(4) Recurrences are affected by the possible presence of low frequency trends (as also noticed in Pincus and Goldberger, 1994). Indeed, similar patterns of activity are not identified as recurrences if they are biased by a trend that translates them around different mean values (with difference larger than *r*). Thus, the use of a high-pass filter with cutoff related to the duration of the epoch is suggested to remove low frequency trends. Specifically, if the duration of the epoch is *T* seconds, the minimum frequency of a sinusoid that could be represented is 1/*T* Hz. However, more recurrences should be studied to assess statistically the predictability of low frequency oscillations. Thus, the minimum frequency of oscillations that could be reliably explored considering the available epoch (related to the cutoff frequency of the high-pass filter to be used to remove the trend) is *f*_cutoff_ = R/*T*, where *R* is the minimum number of times that a recurrent behavior should be investigated (e.g., *R* = 5–10). However, notice that the removal of trends could be admissible only under some assumptions on the data. For example, it is reasonable if the signal is self-similar (Mandelbrot, 1982, so that the same behavior can be found at different scales, as in the case of fractal dynamics) or if different frequency components are independent (e.g., low frequency movement artifacts in bioelectric signals or different EEG rhythms). On the other hand, there could be conditions in which low frequency trends affect the behavior of other components; indeed, they could be related to a slow change in the state of the system generating the time series, which could affect the patterns of interest. In such cases, low frequency trends cannot be removed and could be studied only if the investigated epoch is extended. This could be admissible only if the data are stationary within it, so that a trade-off arises between the needs of studying reliably low frequency trends (requiring a long epoch) and considering stationary the data (imposing a short epoch).
(5) The values that are close in time are also considered neighbors in the phase space by the algorithm for the estimation of ApEn. This allows to remove possible singularities, i.e., computing the logarithm of zero in equation (3). However, this introduces a bias toward low values of ApEn, as *N*-*m*-1 self-recurrences are always found in both embedding spaces (Pincus and Goldberger, 1994). To remove this problem, Sample Entropy (SampEn) was introduced (Richman and Moorman, 2000): it requires commuting the sum and the logarithm operators in (3) (in addition to avoiding singularities, SampEn is a measure of complexity that is very different from ApEn, as indicated in the “Discussion” section). As an alternative, a simpler way to overcome the problem could be the introduction of a Theiler window (Kantz and Schreiber, 1997), i.e., a time window defining a delay under which points cannot be considered recurrent; then, the points which have no recurrences should be removed from the computation of ϕ^m^(r) (in order to avoid singularities).

### Modified ApEn

Based on the previous observations, the modified ApEn is proposed. The following variations are included.

(1) The time delay τ between coordinates can be different from 1 (it should be taken in linear relation with the sampling frequency, as mentioned above).
(2) The tolerance *r* is chosen in order that a specific percentage (selectable by the user) of recurrence points are found in dimension *m* (in this way, the possibility that too few recurrences are found is avoided). This idea was also considered in the minimum numerator count method (Lake and Moorman, 2011). This allowed to get stable estimations of quadratic sample entropy (SampEn modified to make it stable to a variation of *r*) with very short RR series. The literature proposed also other alternatives, as that of choosing the value of *r* corresponding to a maximal ApEn (or an approximation of it, in order to reduce the computational burden, Lu et al., 2008). This method showed good results in the literature (Lu et al., 2008), even if it didn’t discriminate heart failure and healthy controls from heart rate variability data (Liu et al., 2011). Further advanced techniques have been proposed to select an optimal value of the tolerance based on the asymptotic theory of bandwidth selection for kernel density estimators (Lake, 2010; Darmon, 2016) or building efficiently an ApEn profile using different values of tolerances (Udhayakumar et al., 2017).
(3) The total number of embedded vectors is the same, equal to *N-*(*m*+1)τ, in the two embedding dimensions (*m* and *m*+1), as the last vectors in the lowest dimension are discarded. In this way, the index is always non-negative.
(4) Low frequency trends, which cannot be properly studied due to the finite duration of the epoch, are removed by a high-pass filter with cuttoff proportional to the inverse of the epoch duration.
(5) A Theiler window is introduced (so that points closer in time than a delay, which can be selected by the user, are not considered neighbors; for example, in the datasets considered below, only self-recurrences, corresponding to a delay of 0, were removed). Points in dimension *m* which have no recurrences are not considered; those which have recurrences in dimension *m* and no recurrence in dimension *m*+1 are modified by adding one recurrence to the correlation integrals (it’s like assuming to add a further recurrence which does not lead to divergence of the trajectory, or considering self-recurrences only for these problematic points).

## Results

**FIGURE 1:**
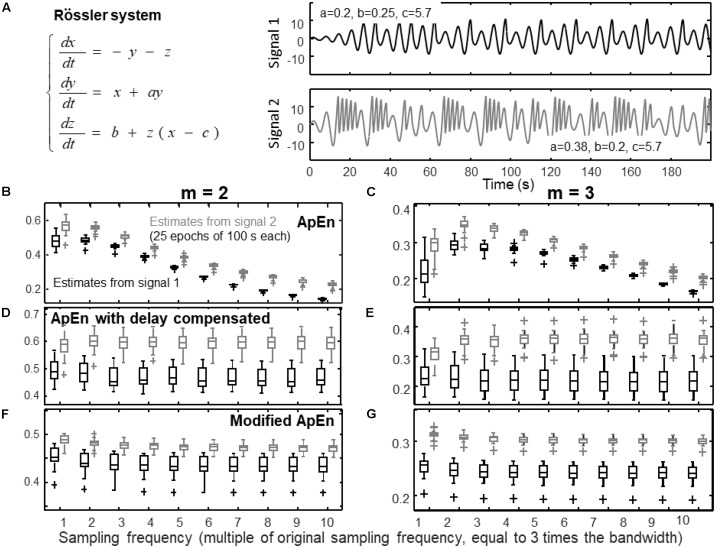
Estimation of complexity indexes of signals with different sampling frequencies. **(A)** Definition of the Rössler system used to simulate a pseudo-periodic and a chaotic signal (signals 1 and 2, respectively, are the components *x(t)* of the system for the two indicated sets of parameters). **(B)** Estimates of ApEn of 25 epochs of signals (median, quartiles, range and outliers shown individually) with embedding dimension *m* = 2 (*r* = 0.2 std). **(C)** Estimates of ApEn with *m* = 3. **(D)** Estimates of the entropy considering the same algorithm used for ApEn, but using a delay τ proportional to the sampling frequency and *m* = 2. **(E)** Same as **(D)**, but with *m* = 3. **(F)** Modified index: self-recurrences removed, delay τ proportional to the sampling frequency, percentage of recurrence points for the lowest dimension imposed to be the 20% of the number of samples of the time series, *m* = 2. **(G)** Same as **(F)**, but with *m* = 3.

**FIGURE 2:**
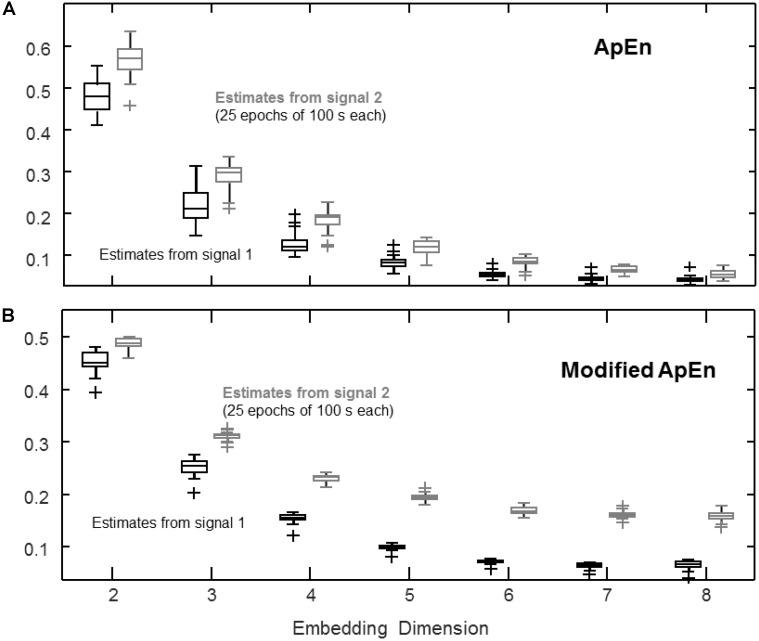
Estimation of complexity indexes considering different embedding dimensions *m*. The same signals as in **Figure 1** are considered, sampled at three times the bandwidth of the signals. **(A)** ApEn (median, quartiles, range and outliers shown individually) estimated for 25 epochs of 100 s (*r* = 0.2 std). **(B)** Same data as in **(A)**, processed by the modified index: self-recurrences removed, τ = 1, percentage of recurrence points for the lowest dimension imposed to be the 20% of the number of samples of the time series.

**FIGURE 3:**
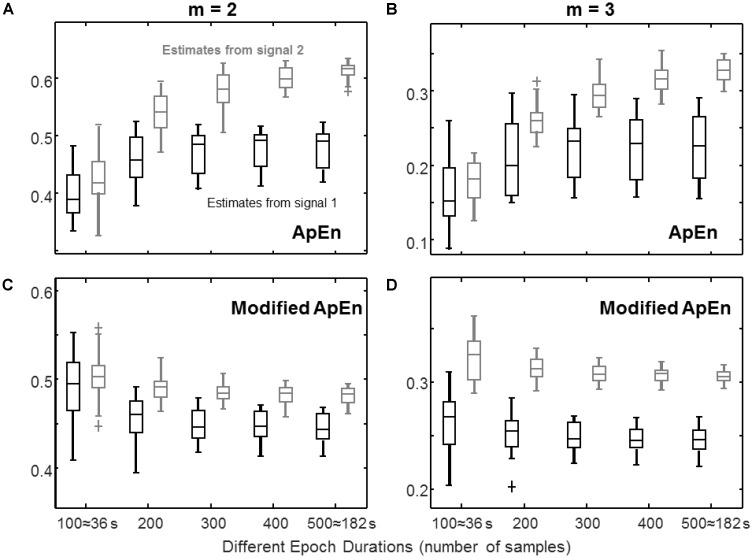
Estimation of complexity indexes from epochs of different durations. The same signals as in **Figure 1** are considered, sampled at three times their bandwidth. **(A)** ApEn (median, quartiles, range, and outliers shown individually, for 25 different epochs) estimated for epochs of different durations with *m* = 2 (*r* = 0.2 std). **(B)** Same as in **(A)**, but with *m* = 3. **(C)** Same data as in **(A)**, processed by the modified index (with *m* = 2): self-recurrences removed, τ = 1, percentage of recurrence points for the lowest dimension imposed to be the 20% of the number of samples of the time series. **(D)** Same as **(C)**, but with *m* = 3.

**FIGURE 4:**
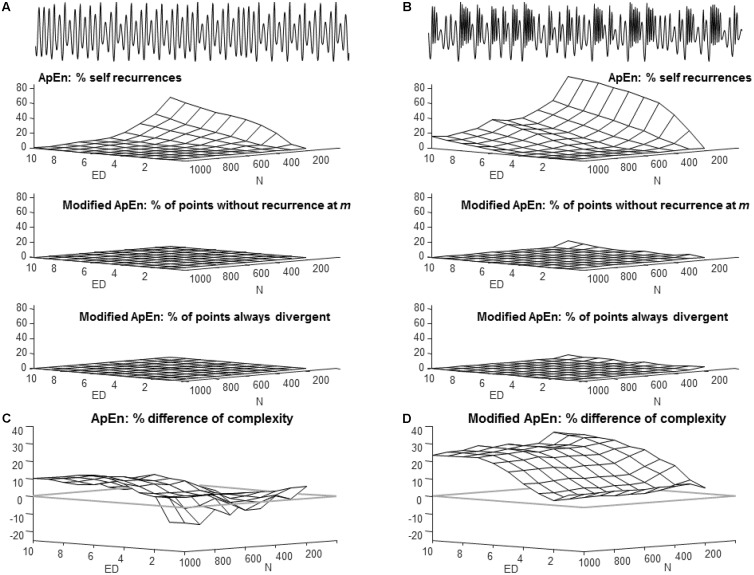
Points without matches and their impact on complexity estimation. **(A)** The same regular signal considered in **Figure 1** (called signal 1) and sampled at 3 times the bandwidth is processed, considering different embedding dimensions ED and numbers of samples *N* (a single epoch was considered for each test). The following indications are provided (percentages of the total number of processed samples): points with only self-recurrence in ApEn estimation (*r* = 0.2 std), points without recurrences for modified ApEn for ED = *m* (total number of recurrence imposed to be 20% of *N*), points in which trajectories are always divergent (i.e., never recurrent for ED = *m*+1, considering the threshold chosen for the estimation of the modified ApEn). **(B)** Same as **(A)**, but for the chaotic signal considered in **Figure 1** (signal 2). **(C)** Percentage difference of the ApEn of the chaotic and regular signals (positive number expected). **(D)** Same as **(C)**, but considering the modified ApEn.

**FIGURE 5:**
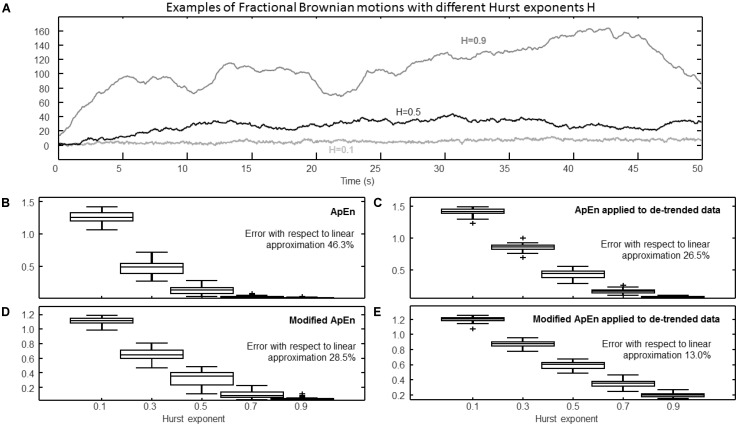
Estimation of complexity indexes considering fractional Brownian motions (fBm) with different Hurst exponents. **(A)** Epochs of different signals (they were assumed to be sampled at 100 Hz). Data may include slow trends: in the following panels, the effect is shown of either keeping or removing the trend (by a high-pass filter of Chebychev Type II, order 4, with cutoff 5/*T*, where the duration of the processed epochs was *T* = 50 s). **(B)** ApEn (median, quartiles, range, and outliers shown individually) estimated for 25 epochs of fBm with Hurst exponent ranging from 0.1 to 0.9 (*r* = 0.2 std). The mean values of the estimates versus Hurst exponents were interpolated by a line and the percentage root mean squared error (with respect to the overall mean) is indicated. **(C)** Same as **(B)**, but considering de-trended data. **(D)** Modified ApEn (self-recurrences removed, τ = 1, percentage of recurrence points for the lowest dimension imposed to be 5%) applied to raw data. **(E)** Modified ApEn applied to de-trended data.

**FIGURE 6:**
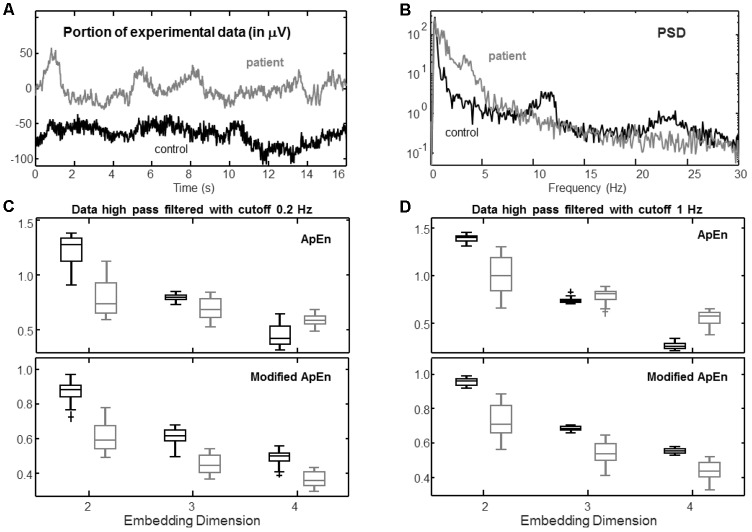
Example of experimental application. Two EEGs were recorded in rest conditions from a healthy control and a patient who entered a vegetative state with closed eyes (duration of the recording 250 s, sampling frequency 256 Hz; before processing, data were resampled at 100 Hz after low-pass filtering with cutoff 32 Hz using a Chebyshev Type 2 filter of order 13). Data from channel F7-RF are considered. **(A)** Example of a portion of data from the two subjects. **(B)** Power spectral density (PSD; estimated by the Welch method, using Hamming windows of duration 16 s with no overlap). **(C)** Distribution of estimations of ApEn (*r* = 0.2 std) and modified ApEn (self-recurrences removed, τ = 1, percentage of recurrence points for the lower dimension imposed to be 10%) considering 25 epochs of 8 s, high-pass filtered with cutoff 0.2 Hz (Chebyshev Type 2 filter of order 4). **(D)** Same as **(C)**, but considering a high-pass filter with cutoff 1 Hz (Chebyshev Type 2 filter of order 6).

ApEn and the modified index were tested on simulations (**Figures 1**–**5**) and experiments (**Figure 6**). Representative examples are considered for which the reliability of ApEn is pushed to the limit. Referring to the section “Materials and Methods”, in the following the embedding dimension is indicated with *m* and the threshold with *r*.

### Effect of Data Over-Sampling

**Figure 1** shows the effect of over-sampling the signals on ApEn estimation. The system of Rössler (1976) was used to simulate two time series (corresponding to different sets of parameters, as indicated in **Figure 1A**): a quasi-periodic signal and a chaotic one. These two time series are simple prototypes of a predictable and a complex signal, respectively, and are considered also for the following **Figures 2**–**4**. The frequency range including both signals was explored, computing the bandwidth allowing to preserve 99% of the energy (which is about 0.9 Hz). Then, the data were resampled at different frequencies: the minimum frequency was three times the bandwidth, the others were multiple of such a sampling rate.

As mentioned in the section “Materials and Methods”, by increasing the sampling frequency, the time series become more predictable, as the dynamics is linearized. Indeed, **Figures 1B,C** show that ApEn decreases for large values of the sampling frequency. Notice that 30 times the bandwidth is the maximal sampling frequency considered here, but, as mentioned in the section “Introduction”, in the literature there are even examples of papers in which the sampling frequency was around 100 times the bandwidth. By changing the delay of the phases according to the variation of the sampling frequency as in **Figures 1D,E**, the effect of linearization is compensated, so that the estimates are more stable. The modified index (including also self-recurrence removal and a fixed percentage of neighbors) is considered in **Figures 1F,G**. Signal discrimination is very good with *m* = 3 (**Figure 1G**), i.e., considering an embedding dimension equal to the number of state variables of the Rössler system. In this way, false recurrences (or false near neighbors) due to projection to a low dimensional space are removed and the trajectory of the system can be correctly explored (Kantz and Schreiber, 1997; Mesin, 2017).

Notice that, in this specific application, the discrimination capability of ApEn is increasing by over-sampling the data, which could be considered as a positive effect. However, in the considered case, the bandwidths of the two signals were similar. Consider instead the case of comparing a regular signal of high frequency and a complex one of low frequency: it could be explored in **Figure 1** by comparing an over-sampled signal that is complex (signal 2) and a quasi-periodic signal sampled at a lower rate (signal 1). In such a case, the discrimination of the two signals would be hampered^2^. Moreover, over-sampling could be prone to high frequency noise, as mentioned in the section “Materials and Methods.”

### Effect of Embedding Dimension

**Figure 2** shows the effect of increasing the embedding dimension. The same signals studied in **Figure 1** are considered with a sampling frequency of three times the bandwidth. The value of ApEn shown in **Figure 2A** decreases as the embedding dimension increases, as the number of recurrences drops and the bias due to self-recurrences leads to low values of ApEn for both signals. On the other hand, by imposing a fixed percentage of recurrences in dimension *m*, some discrimination capability is preserved, as shown in **Figure 2B** (self-recurrences were also removed; as a result, the index is not biased toward 0).

### Effect of Epoch Duration

**Figure 3** shows the stability of complexity estimation when the epoch duration is reduced. With short epochs, the number of recurrences decreases when keeping constant the tolerance *r*. The value of ApEn is then affected by the bias introduced by self-recurrences (reflected in a lower value of estimated complexity) and by the predictability of rare events. Rare recurrences are poorly represented, but have a large weight due to the logarithm function present in the definition of ApEn, so that they induce large variability. The modified ApEn does not consider self-recurrences and imposes to use always a large number of recurrences for the lowest dimension (in this case, the 20% of the number of samples). The estimates are more stable than those of ApEn. Moreover, for a large enough embedding dimension (i.e., *m* = 3 for this system which has three state variables), the modified index allows to discriminate the two signals with short epochs. For example, considering that a perfect discrimination is achieved when the ranges of the estimates obtained from the two signals are separated, ApEn shows this condition only for epochs of at least 400 samples with *m* = 2 (or 500 samples with *m* = 3), whereas the modified index discriminates perfectly the two signals with *m* = 3 using epochs of 200 samples (or longer).

### Impact of Self-Matches

**Figure 4** allows to deepen the effect of the choice of the embedding dimension (already considered in **Figure 2**) and of the duration of the epoch (**Figure 3**), showing the number of templates without recurrences, in order to give insight on the impact of self-matches. The same simulated signals as in **Figure 1** are considered. ApEn has a large percentage of points without any match, apart from the self-recurrence (**Figure 4A**): these points bias the estimation toward low values. There is an increasing trend of templates without matches when the embedding dimension is increased and the epoch shortens. The effect is larger for the chaotic than for the regular signal (maximal values of self-recurrences are about 82 and 54%, respectively). For this reason, the order of complexity estimated using ApEn is even inverted (with a larger ApEn for the regular signal than the chaotic one), when the embedding dimension is large and the epochs are short, as shown in **Figure 4C**. This problem does not affect the modified index (in **Figure 4D**, the difference of complexity of the chaotic and regular signals is always positive). This reflects the lower bias due to points with no matches, which are only a few when considering the modified ApEn. Indeed, the number of points in dimension *m* without any match are 0 and lower than about 3%, for the regular and chaotic signal, respectively (**Figures 4A,B**; these points are removed from the computation of modified ApEn, as indicated in the section “Materials and Methods”); the points which show always divergence of the trajectory (so that there are recurrences at embedding dimension *m*, but not for *m*+1) are 0 and lower than about 5%, for the regular and chaotic signal, respectively (**Figures 4A,B**; these are the points which are compensated, adding a self-recurrence, as indicated in the section “Materials and Methods”).

### Effect of Low Frequency Trends

**Figure 5** shows the effect of trends and their removal using a high-pass filter (Chebyshev Type II, with 20 dB of minimum attenuation in the stop-band). Realizations of fractional Brownian motion (fBm) were considered, as they show important trends with high values of the Hurst exponent H (**Figure 5A**). The trend affects the identification of recurrences: if it is not removed, the number of recurrences is low and ApEn is largely affected by the bias induced by self-recurrences. Thus, with high values of H, the level of complexity estimated using ApEn is close to zero (as shown in **Figure 5B** for *H* > 0.5). By removing the trend, the signals can be better discriminated (**Figure 5C**). The modified ApEn (considered in **Figures 5D,E**) is less affected by the trend (as self-recurrences are removed and many matches are always found); however, it also benefits of the removal of the low frequency trend (as shown in **Figure 5E**). Notice that the fractal dimension *D* of the fBm is linearly related to H (*D* = 2-H): it is interesting that the mean values (across different processed epochs) of the modified ApEn show a close to linear variation with H.

### Representative Application to Experimental EEG

**Figure 6** shows a representative application to EEG data from a healthy subject and a patient who, after a brain trauma, entered a vegetative state. The study was approved by the Ethical Committee of the Hospital CTO (Centro Traumatologico Ortopedico, i.e., Centre for Orthopaedic Trauma) in Turin, conducted following the principles of the Declaration of Helsinki and provided by a medical doctor (acknowledged at the end of the paper). Data were acquired from 19 channels using the standard 10–20 during a rest condition with closed eyes for a duration of about 4 min. Sampling frequency was 256 Hz, but data contained more than 99% of their energy below 30 Hz, so that they were resampled at 100 Hz after low-pass filtering (Chebyshev Type II, order 13, stop-band starting at 32 Hz with 30 dB of minimum attenuation). Channel F7-RF was selected. Notice from **Figure 6A** that the EEG from the patient shows low frequency trends, confirmed by the power spectral density shown in **Figure 6B**. The data from the healthy subject contain larger components of high frequency, e.g., there is a peak in the alpha band and some contribution in beta.

Data were split into 25 epochs of 8 s. Different complexity indexes were computed after high-pass filtering with a cutoff of either 0.2 or 1 Hz (Chebyshev Type II, with 20 dB of minimum attenuation in the stop-band). In this way, movement artifacts were removed; moreover, when attenuating components under 1 Hz, trends that cannot be reliably studied in the considered epochs were attenuated.

Data were compared with surrogates (Mesin, 2017). Specifically, for each processed epoch, 20 surrogates were generated using the iterative amplitude-adjusted Fourier transform method (Schreiber and Schmitz, 1996). These surrogates had the same amplitude distribution and (approximately) the same spectral density of the original data, but they were stochastic. For more than the 80 and 88% of the epochs, ApEn and the modified index (respectively) were significantly lower than those computed from surrogates (Wilcoxon rank sum test, significance level 0.05). The number of statistically significant cases was higher for the data from the healthy subject, as they were more complex (so that it was easier for a test of non-linearity to indicate the significance).

The modified index allows to get a better discrimination of the two cases. It appears to be evident from the graphs, but it is also confirmed when measured by the Fisher discrimination ratio (Theodoridis and Koutroumbas, 2008), which was in the average about 3 and 5 for ApEn and the modified index, respectively. Moreover, the modified ApEn provides consistent results in all conditions, indicating that the data recorded from the healthy subject is more complex than the EEG from the patient (in line with the literature, which indicates that EEG complexity decreases if the level of consciousness is lower; Burioka et al., 2005; Koskinen et al., 2006; Wu et al., 2011). On the other hand, the standard ApEn provides different indications, depending on the choice of parameters and on the de-trending filter used.

Notice that the embedding dimensions (estimated using the Cao’s method, Cao, 1997) were equal to 4 for both signals (so that false recurrences were completely removed when considering *m* = 4). Indeed, in such a case, the modified index allows to get a good discrimination of the two signals and reliable indications of complexity (in contrast with the estimations of ApEn with the same value of *m*).

## Discussion

S. M. Pincus indicated that ApEn should be considered only in relative sense (Pincus and Goldberger, 1994), after fixing the values of embedding dimension *m* and tolerance *r*. However, standardizing the choice of parameters would help in comparing different studies. Stressing this concept is important as very different values of parameters can be found in the literature, even when the same type of data is studied (here, EEG was considered). Moreover, some studies were conducted considering parameters for which the estimation of ApEn, as a statistical index of complexity, was not reliable (see the “Introduction”, where examples of studies are given in which the embedding dimension was larger than 5 and the sampling frequency was more than 10 times the bandwidth of the considered signal).

### Problems in ApEn Estimation and Proper Choice of Parameters

This work provides some examples of simple conditions in which ApEn gives not stable indications. Specifically, ApEn is affected by the sampling frequency of the processed data, the embedding dimension, the duration of the processed epoch and the presence of low frequency trends. The behavior of ApEn when varying each of these parameters can be interpreted as follows:

(1) Oversampling induces a linearization of the deterministic information contained into the data;
(2) Increasing the embedding dimension reduces the probability of finding recurrences, making ApEn estimation less stable and affected by self-recurrences;
(3) The shortening of the processed epoch reduces the time span in which the dynamics is explored, with the risk of failing to observe specific behaviors or to find recurrences;
(4) Low frequency trends affect the estimation of recurrent patterns.

These observations provide some indication on how to choose parameters in order that ApEn can give reasonable indications and that different studies can be compared. In particular, the following suggestions are given.

(1) The sampling frequency should be close to the Nyquist limit, otherwise, for larger sampling rates, the dynamics of the system generating the time series is linearized and the value of ApEn decreases (because the next sample is more predictable, as it is close to the previous ones; see **Figure 1**), unless a high frequency noise is present, so that its complexity biases the estimation of ApEn to large values. The bias in ApEn estimation due to the sampling frequency can hinder the comparison of studies on similar data sampled at different rates.
(2) Low values of embedding dimension should be used (as already suggested in Pincus and Goldberger, 1994). By increasing the embedding dimension, recurrences are harder to be found (see **Figure 4**), so that self-recurrences have a greater influence in biasing ApEn toward low values (**Figure 2**). Moreover, it is more difficult to discriminate the signals with different levels of complexity considered in **Figure 2** for larger embedding dimensions. However, more recurrences can be found by increasing the sampling frequency, so that there could be a combination of parameters, with high embedding dimension and sample rate, for which ApEn provides results which could appear interesting (either because they are in line with the literature or as they allow to discriminate better among different groups). This paper does not support to keep these apparently good outcomes, as they result from two wrong choices of the parameters. In fact, an embedding space with too large dimension cannot be explored by the short epochs which are usually processed and a sampling rate which is too high has the effect of linearizing the dynamics of the investigated system (moreover, the complexity of the high frequency noise could further bias the estimation of ApEn, as mentioned above). The interpretation of the results are further complicated by the different possible choices of the threshold *r*. Indeed, by increasing its value, runs that are more distant are considered as neighbors, increasing the number of recurrences. Thus, it is another parameter that could compensate for the reduced number of recurrences when using a large embedding dimension. This could allow to get again (by chance) some good outcomes, when searching for optimal results by making an exhaustive test of all combinations of parameters. However, by using a too large value of *r*, far runs are considered neighbors, so that the possible divergence of distant points is studied to compute ApEn, even if it is not an indicator of complexity (only the divergence of close points indicates system complexity). Thus, for a reasonable estimation of ApEn, the tolerance should be fixed at a small value relative to std, as suggested in (Pincus and Goldberger, 1994). Notice that in the literature there was even the attempt of fixing the value of the threshold, without reference to std: this choice should be avoided, as in such a case ApEn would obviously depend on the signal amplitude (Sarlabous et al., 2010); this was even considered as a positive outcome, though there are better solutions to estimate the signal amplitude than using an index of complexity!
(3) The epoch duration should be chosen as a trade-off between the need of considering the signal stationary and that of providing enough information to decode the dynamics of the system. Shortening the epochs, the estimation of ApEn is more and more unstable, as fewer recurrences are found (see **Figure 4**), so that the dynamics of different neighbors are not well represented to extract statistically stable conclusions about convergence or divergence of trajectories. **Figure 3** shows the instability of the estimations of ApEn as the epoch duration shortens. **Figure 4** indicates also that, using a large embedding dimension and short epochs, ApEn could be even larger for regular than for chaotic data (thus, arbitrary indications could be obtained by changing parameters out of reasonable ranges, as mentioned above).
(4) Removing the low frequency trend is important (**Figure 5**), even if it is allowed only under specific assumptions (i.e., if the dynamics at shorter temporal scales is not affected by the trend, as discussed in the section “Materials and Methods”). The epoch duration imposes the minimum frequency of oscillations that can be studied (at least 5–10 low frequency oscillations should be available in the epoch in order to explore possible recurrences); it is also related to the time interval in which the data can be considered stationary, as mentioned above.

### Properties of the Modified ApEn

The previous observations suggest modifying ApEn in order to get more stable indications in some critical conditions: a new index was then proposed, which was here called modified ApEn. The main variations with respect to the standard index are the followings: a delay between phases (possibly different from 1) is considered to compensate for an over-sampling, self-recurrences are avoided by introducing a window within which recurrences are removed and the threshold is defined imposing a fixed percentage of neighbors for the trajectory in dimension *m* (the number of recurrences will drop in dimension *m*+1, depending on the level of complexity). The results of this paper do not guarantee that this new index is always to be preferred with respect to ApEn. Indeed, in some conditions, requiring a fixed number of recurrences could result in a large threshold, so that there could be distant points considered as neighbors (the correct percentage of recurrences should be selected in order that the threshold is feasible, otherwise neighbors could be too distant). Moreover, only representative examples of data are here considered and with specific values of the parameters (a test on a large range of conditions and the comparison of different methods for complexity estimation is beyond the aims of this work, but an interesting topic for future investigation). Thus, there could be conditions, which were not found here, in which the new index gives unstable or contradicting results. However, the new algorithm was developed under reasonable hypotheses and, at least for the limited cases considered in this paper, it provided some interesting results. For example, the following outcomes are of relevance.

(1) The new index compensates for over-sampling (as shown in **Figure 1**), providing an indication of complexity which is not related to the specific sampling rate or to the bandwidth of the data (whereas ApEn has a bias toward higher values for data containing components of higher frequency, independently of their level of regularity).
(2) The modified ApEn allows to get reasonable estimates even for large values of the embedding dimension. Indeed, the number of recurrences does not drop as *m* increases, as in the case of the standard index. This allows to the modified index to maintain a good discrimination of the simulated data with different levels of complexity shown in **Figure 2**^3^.
(3) The new index, when using a proper embedding dimension, allows to obtain stable results with shorter epochs than the standard ApEn, as shown in **Figure 3** (again, this is due to the large number of recurrences that are always available, even with short epochs).
(4) When applied to fractional Brownian motion after low frequency trend removal (**Figure 5**), the modified ApEn linearly scales with the Hurst exponent (and hence also with the fractal dimension) of the simulated data.

Finally, the two indexes were applied to representative examples of EEG from a patient in vegetative state and a healthy control. ApEn showed contradicting results considering different sets of parameters, whereas the modified version indicated that the complexity was always larger for the data from the control subject (in line with the literature, Burioka et al., 2005; Koskinen et al., 2006; Wu et al., 2011). Moreover, good discriminability was obtained only for the modified index when using *m* = 4, which is the correct embedding dimension (i.e., the one guaranteeing to remove false recurrences). This indication, together with those obtained from simulations, suggests that the correct embedding should be used (but only with the modified ApEn), as it allows to correctly identify recurrences.

### Comparison With SampEn

Additional results are shown in the Supplementary Material, where the modified ApEn is compared to SampEn, which is another popular index for estimating complexity in biomedical data, already introduced to compensate for some problems in the use of ApEn (Richman and Moorman, 2000). It measures the Renyi entropy rate of order 2 or quadratic entropy rate, whereas ApEn estimates the Renyi entropy rate of order 1 (Lake, 2010). It does not require to include self-recurrences and is more stable, as it is not affected by the local behavior of the time series, which could be poorly represented in short time series, as in the case of rare events. Notice that this is a strength of SampEn, but also a limitation, as this index reflects the global regularity, without emphasizing rare events, i.e., those that are more informative. However, there are conditions in which irregularity is ruled by rare events. For example, some systems show bursts of activities (Keener and Sneyd, 1998), which reflect the transition between a few states and only the times of transition between them, not the dynamics within them, are irregular. Also EEG could be interpreted as an alternation of regular rhythms (with the inclusion of some additional particular waveforms), where most of the irregularity is due to the schedule of the transitions between them. As shown in the Supplementary Material, SampEn is affected by sampling rate, embedding dimension, duration of the processed epoch and low frequency trends, with results similar to those of ApEn. However, consider that the classical algorithm for SampEn estimation was considered, but more stable methods have been introduced in the literature (Lake and Moorman, 2011). Its classical definition could also be modified in the future, with a straightforward integration of the ideas discussed here (e.g., possible over-sampling can be compensated and low frequency trends can be removed).

### Other Advanced Complexity Measurements

In this paper, the standard algorithms to estimate ApEn and SampEn were considered to be compared to the proposed modified ApEn. As already mentioned, other approaches were proposed in the literature to get better estimations of complexity, in terms of robustness and consistency in short epochs.

For example, an interesting method to estimate complexity in very short epochs is given in (Lake and Moorman, 2011), where a variation of SampEn is considered. The same problem was faced in Udhayakumar et al. (2017), where a profile of ApEn values over different tolerances was computed, from which secondary measures were extracted to estimate complexity. Another approach is provided by the corrected conditional entropy (CCE), discussed in Porta et al. (1998). It requires computing the minimum with respect to the embedding dimension *m*, avoiding the selection of a fixed pattern length. Computing the maximum of ApEn with respect to the threshold *r* was proposed in Lu et al. (2008). The last two methods appear to be computationally intensive, as different values of either *m* or *r* should be considered.

Maybe, for their computational cost or because of their complex interpretation, the above-mentioned methods had limited outcome in the literature on applications, compared to the classical ApEn and SampEn. On the other hand, the modified ApEn has the same interpretation as the original ApEn upon which it is built. Moreover, it has a computational cost that is similar to that of ApEn and SampEn (tests on data with a number of samples among 100 and 3000 indicates that the computational cost of the present implementation^4^ of the modified ApEn is twice that of classical algorithms).

## Conclusion

ApEn is a complexity index, which was extensively used (and maybe also abused) in the literature. It is sensitive to the choice of parameters that sometimes were chosen out of reasonable ranges. Thus, different results could be obtained by widely changing parameters, when looking for an indication that better fits the expectation of the user. However, this is not a correct way for searching complexity in the data. This paper shows some simple conditions in which ApEn provides unstable or contradicting results and proposes a modification of the index, which could compensate in part for these problems. The advice is to use ApEn or its modified version with caution, selecting the parameters in order that the estimations are in line with the rationale behind the definition of such indexes.

## Author Contributions

The author confirms being the sole contributor of this work and approved it for publication.

## Conflict of Interest Statement

The author declares that the research was conducted in the absence of any commercial or financial relationships that could be construed as a potential conflict of interest.

## Footnotes

1 Notice that, by making many tests, it is possible to obtain statistically significant results even from random numbers. For example, using a level of significance of 5%, in the average there is 1 trial out of 20 which comes to be significant by chance. Thus, by making many tests with different combinations of *m*, *r*, and *T*, significant results could come out by chance, because of over-fitting of the data. This could justify the difficulty in replicating some results or the contradicting outcomes of some studies.

2 Notice that, by changing the sampling rate as suggested, also the number of samples would vary. Some tests were performed to check that indeed the estimation of complexity would be hampered even keeping constant the duration of the epoch. Epochs of 1000 samples of the two signals were considered. Signal 1 was scaled to get a bandwidth 50% larger. In this conditions, ApEn estimated a larger complexity for signal 1 (quasi-periodic) than for signal 2 (chaotic) both for *m* = 2 and *m* = 3, considering a sampling frequency of 8 times the bandwidth of signal 2.

3 This property could allow to estimate the modified ApEn on data correctly embedded (i.e., using a dimension which guarantees to remove false near neighbors, Kantz and Schreiber, 1997). Indeed, the results shown in **Figures 1**–**3** indicate that good discrimination was obtained applying the modified ApEn to data with embedding dimension equal to the number of state variables of the Rössler system. Moreover, **Figure 6** shows good outcomes when applying the modified ApEn with *m* = 4, which is the estimated embedding dimension of the EEGs.

4 The additional computational cost is due to the estimation of the tolerance. It was performed by a dichotomic search on the vector of distances between pairs of points.
