# Supplementary material for: Estimation of Complexity of Sampled Biomedical Continuous Time Signals Using Approximate Entropy
Source: Front Physiol. 2018 Jun 11;9:710. doi: 10.3389/fphys.2018.00710 (PMC6004374; doi:10.3389/fphys.2018.00710)
Supplement: Supplementary file 1 [file Presentation_1.pdf]

Supplementary Material

Estimation of Complexity of Sampled Biomedical Continuous Time Signals using Approximate Entropy

Luca Mesin*

*** Correspondence:** Luca Mesin, luca.mesin@polito.it

Results in addition to those shown in the paper are here presented. The same figures as those shown in the paper are considered, but comparing the modified ApEn with another classical index of complexity: the sample entropy (SampEn).

Figure I shows the effect of over-sampling the signals. The same data were used as those considered in Figure 1 of the main part of the paper (signals simulated using a Rössler system with different parameters). The results concerning the modified ApEn are shown, for an easier comparison. Over-sampling has the effect of linearizing the data, so that SampEn decreases as the sample rate increases. The modified ApEn compensates for the effect of over-sampling, as the delay between phases is increased linearly with the sampling frequency. In this way, its estimates are more stable (i.e., they are similar when considering different sampling rates). Notice that also for SampEn, as in the case of ApEn, the discrimination of the two signals (e.g., as measured by the Fisher discrimination ratio) is better when the sampling rate is increased. However, as noticed in the Results section of the paper, this figure indicates that problems are expected when comparing a regular signal with high frequency components and chaotic data with smaller bandwidth.

Figure II shows the effect of increasing the embedding dimension m, considering the same signals studied in Figure I with a sampling frequency of 3 times the bandwidth. The value of SampEn decreases as the embedding dimension increases, as the number of recurrences drops. Compared to ApEn, the bias due to self-recurrences is avoided, so that SampEn does not go to zero for large values of m. However, its discrimination capability is degraded when the embedding dimension increases, whereas it is preserved by the modified ApEn, as it imposes a fixed percentage of recurrences.

Figure III shows the effect of epoch duration on SampEn and modified ApEn. With short epochs, the number of recurrences considered by the algorithm estimating SampEn decreases. This is reflected into a larger variability of the estimates and a lower discrimination capability. Compared to ApEn, SampEn is not affected by self-recurrences, so that it is not biased toward lower values for shorter epochs. Moreover, it discriminates better the two signals with short epochs (as already shown in Richman and Moorman, 2000). For example, perfect discrimination (i.e., separated ranges of the estimates obtained from the two signals) is achieved by SampEn for epochs of at least 400 samples both with m=2 and m=3 (whereas ApEn needed 500 samples with m=3 to perfectly discriminate the two signals, see Figure 3 of the paper). The modified index, when using the correct embedding dimension (m=3, i.e., the number of state variables of the Rössler system), discriminates perfectly the two signals using epochs of 200 samples (or longer).

Figure IV shows the estimation of the complexity of fractional Brownian motions (fBm). When considering high values of the Hurst exponent H, the data contain important low frequency trends. These trends prevent the identification of recurrences. For this reason, SampEn is close to zero when $H>0.5$ and the low frequency trend is kept. By removing the trend, the signals can be better discriminated. The modified ApEn is less affected by the trend and its average estimates (over different epochs) show a close to linear variation with H.

Figure V shows a representative application to EEG data from a healthy subject and a patient in vegetative state (same data as in Figure 6 of the main part of the paper).

Data were compared with surrogates generated using the iterative amplitude-adjusted Fourier transform method. For more than the 79% and 88% of the epochs, SampEn and the modified index (respectively) were significantly lower than those computed from surrogates (Wilcoxon rank sum test, significance level 0.05). The number of statistically significant cases was higher for the data from the control, as they were more complex.

The data were studied after removing the low frequency trend under either 0.2 or 1 Hz: only in the second case, the residual components of lowest frequency could be reasonably studied in the considered epochs (which were 8 s long, so that, by removing the trends under 1 Hz, the residual components could be explored at least for 8 cycles).

The Fisher discrimination ratio was in the average about 4 and 5 for SampEn, considering the high pass filter at 0.2 and 1 Hz, respectively; the Fisher discrimination ratio was in the average always about 5 for the modified ApEn. In contrast with ApEn, SampEn (as also the modified ApEn) provided consistent results in all conditions, indicating that the data recorded from the healthy subject is more complex than the EEG from the patient.

Reference

Richman JS and Moorman JR 2000 Physiological time-series analysis using approximate entropy and sample entropy, American Journal of physiology. Heart and circulatory physiology **278**(6) H2039-49.

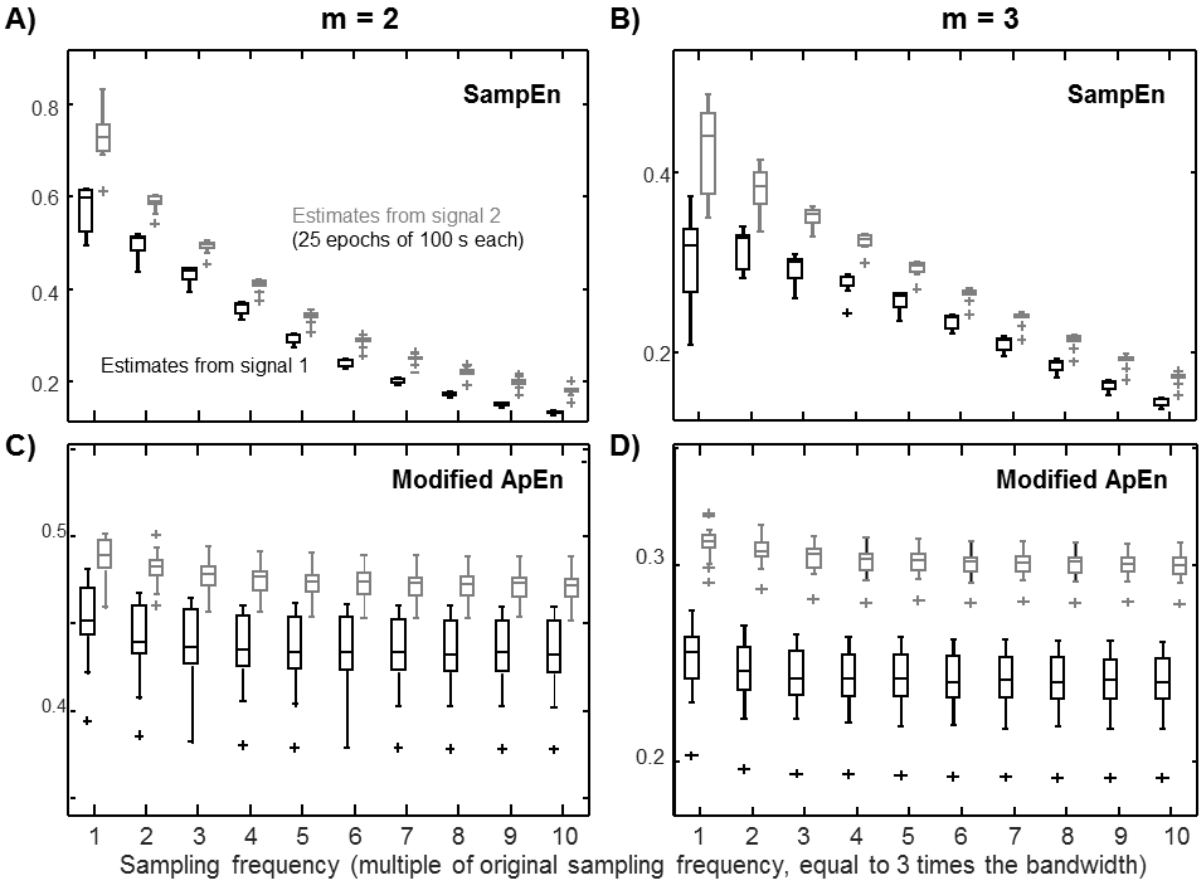

Figure I. Estimation of complexity indexes of signals with different sampling frequencies. The first component of the Rössler system was used to simulate a pseudo-periodic and a chaotic signal (signals 1 and 2, respectively; same data as in Figure 1 of the main part of the paper). A) Estimates of SampEn of 25 epochs of signals (median, quartiles, range and outliers shown individually) with embedding dimension m=2 and threshold r=0.2std. B) Estimates of SampEn with m=3. C) Modified index: self-recurrences removed, delay τ proportional to the sampling frequency, percentage of recurrence points for the lowest dimension imposed to be the 20% of the number of samples of the time series, m=2. D) Same as C, but with m=3.

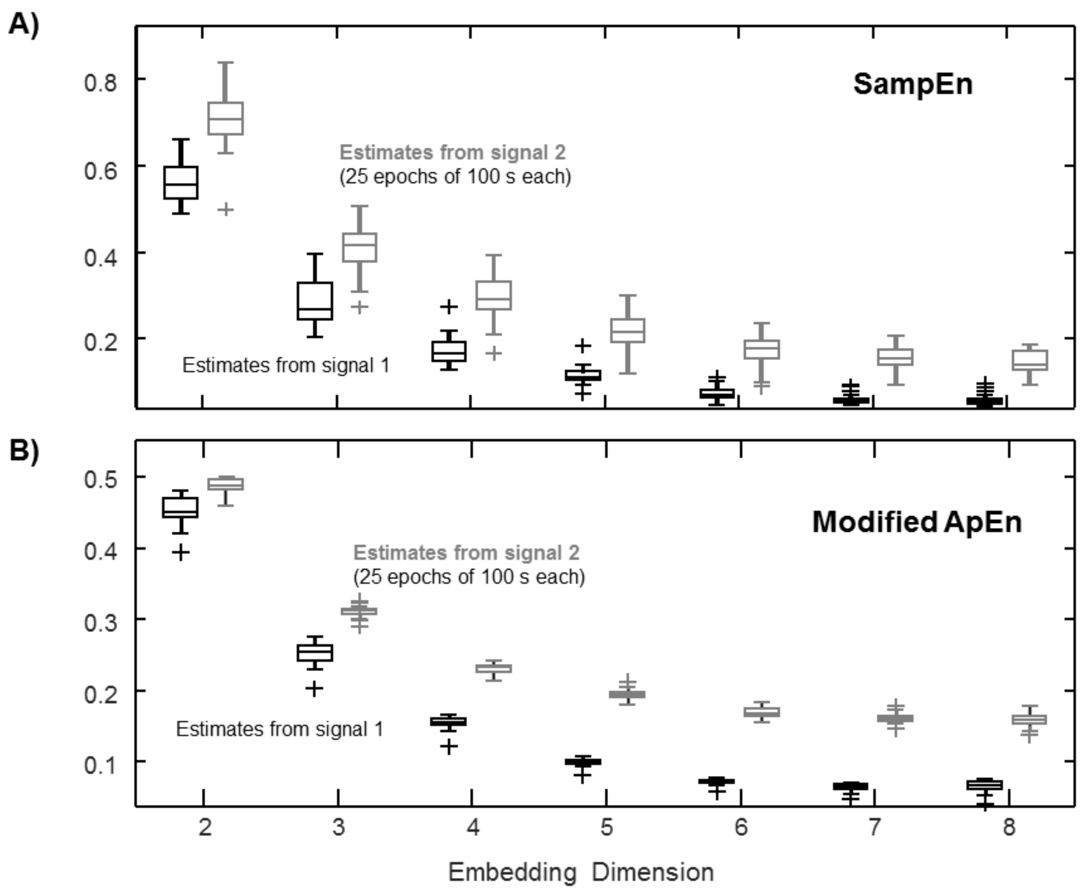

Figure II. Estimation of complexity indexes considering different embedding dimensions. The same signals as in Figure I are considered, sampled at 3 times the bandwidth of the signals. A) SampEn, with r=0.2std (median, quartiles, range and outliers shown individually). B) Same data as in A, processed by the modified index: self-recurrences removed, τ = 1, percentage of recurrence points for the lowest dimension imposed to be the 20% of the number of samples of the time series.

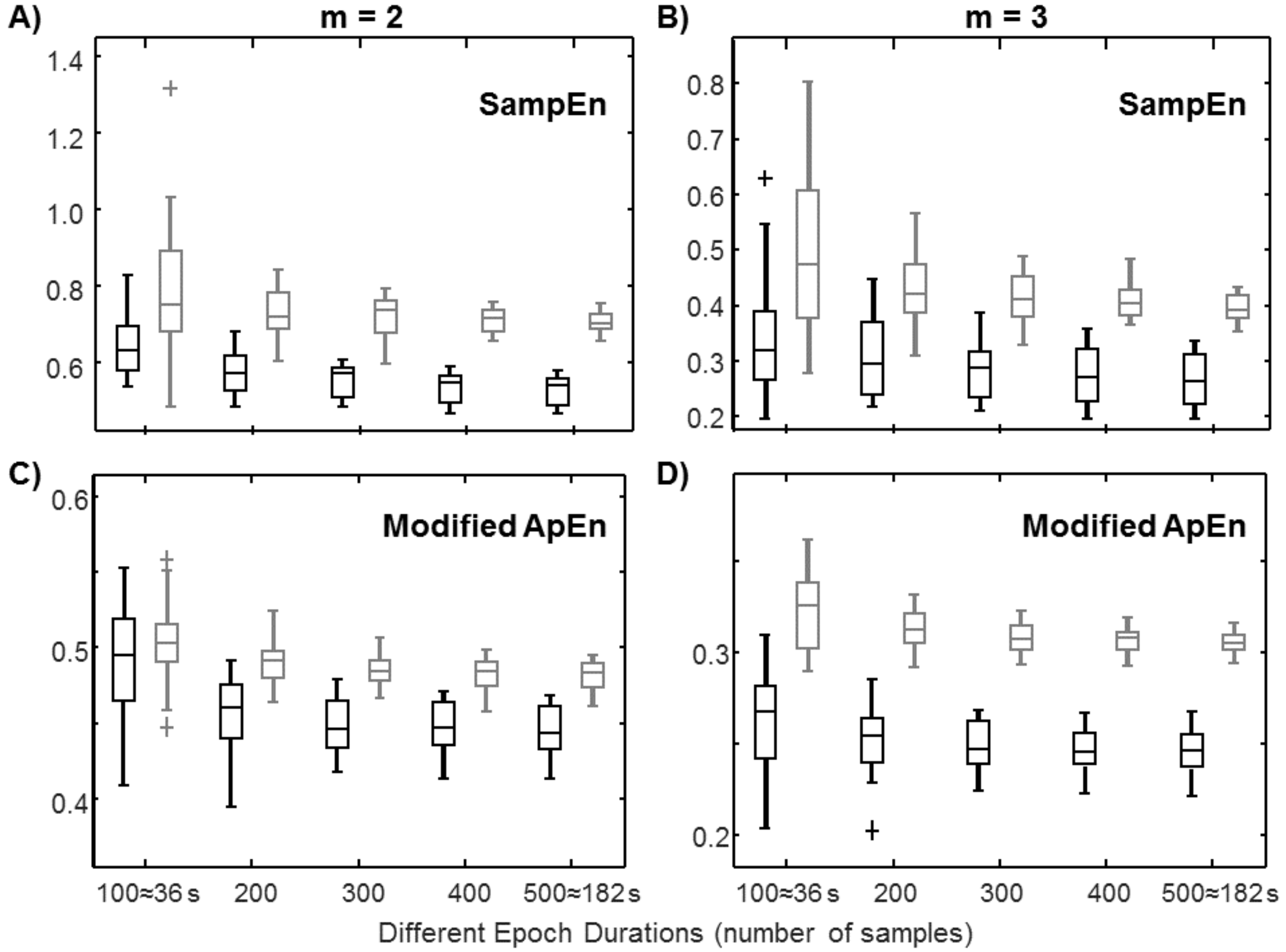

Figure III. Estimation of complexity indexes from epochs of different durations. The same signals as in Figure I are considered, sampled at 3 times their bandwidth. A) SampEn (median, quartiles, range and outliers shown individually, for 25 different epochs) estimated in epochs of different durations with *m*=2 (*r*=0.2std). B) Same as A, but with *m*=3. C) Same data as in A, processed by the modified index considering *m*=2: self-recurrences removed, τ=1, percentage of recurrence points for the lowest dimension imposed to be the 20% of the number of samples of the time series. D) Same as C, but with *m*=3.

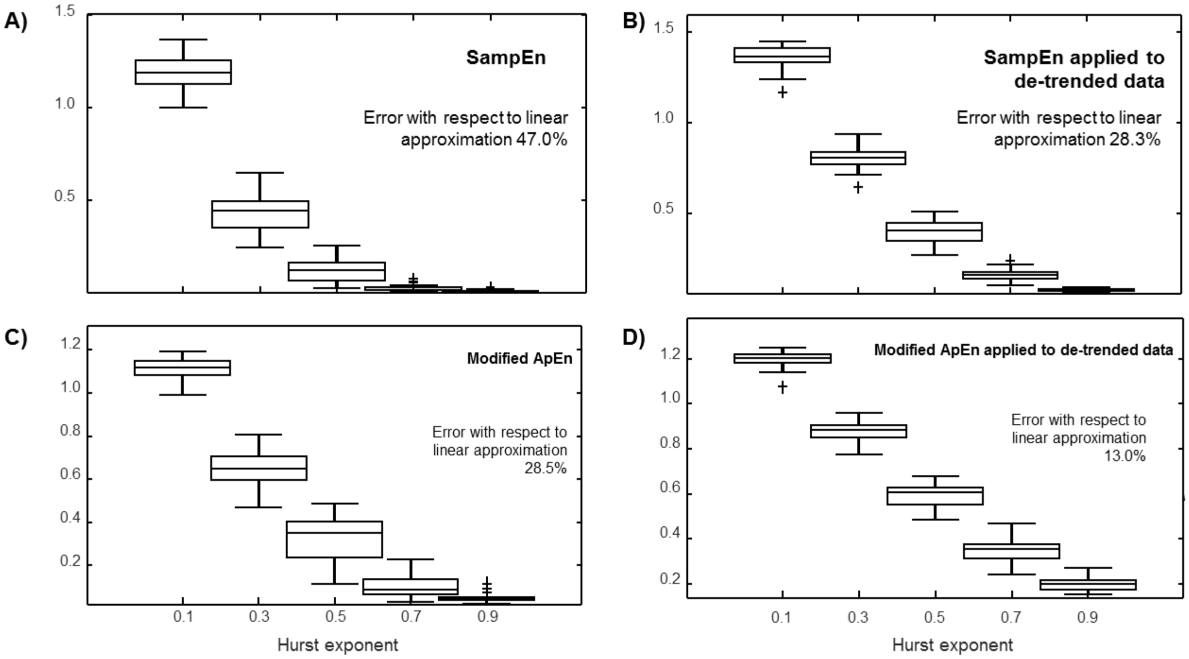

Figure IV. Estimation of complexity indexes considering fractional Brownian motions (fBm) with different Hurst exponents (some examples of data are shown in Figure 5 of the main part of the paper). Data may include slow trends: the effect is shown of either keeping or removing the trend (by a high-pass filter of Chebychev Type II, order 4, with cutoff $5/T$, where the duration of the processed epochs was T=50 s, assuming a sampling rate of 100 Hz). A) SampEn (median, quartiles, range and outliers shown individually) estimated for 25 epochs of fBm with Hurst exponent ranging from 0.1 to 0.9 (r=0.2std). The mean values of the estimates versus Hurst exponents were interpolated by a line and the percentage root mean squared error (with respect to the overall mean) is indicated. B) Same as A, but considering de-trended data. C) Modified ApEn (self-recurrences removed, τ=1, percentage of recurrence points for the lowest dimension imposed to be 5%) applied to raw data. D) Modified ApEn applied to de-trended data.

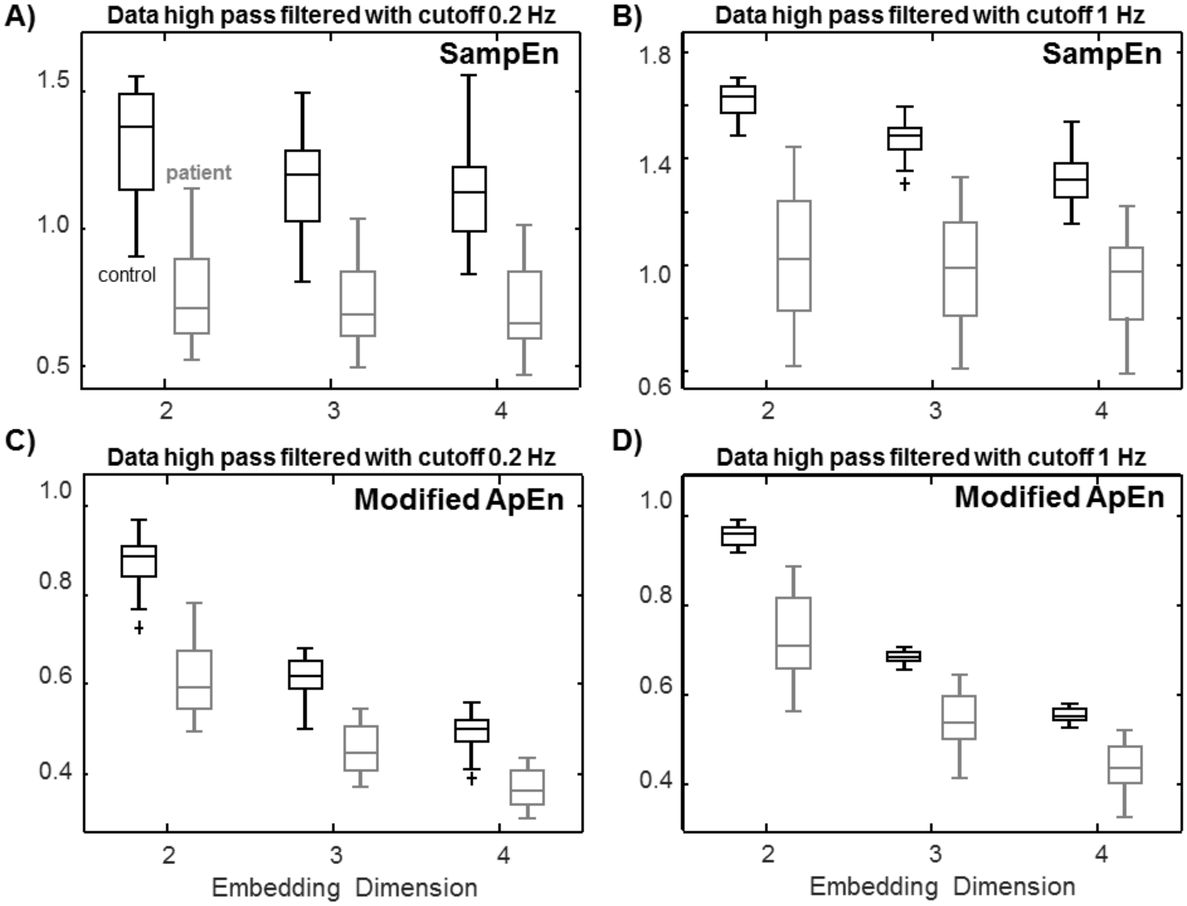

Figure V. Example of complexity estimation of rest EEGs recorded from a healthy control and a patient in vegetative state with closed eyes (same data are considered as for Figure 6 of the main part of the paper and the same filters were used). A) Distribution of estimations of SampEn considering 25 epochs of 8 s, high pass filtered with cutoff 0.2 Hz (r=0.2std). B) Same as A), but considering a high pass filter with cutoff 1 Hz. C) Same as A), but considering the modified index (self-recurrences removed, τ =1, percentage of recurrence points for the lower dimension imposed to be 10%). D) Same as C), but considering a high pass filter with cutoff 1 Hz.
